# Rac1-dependent regulation of osteoclast and osteoblast differentiation by developmentally regulated GTP-binding 2

**DOI:** 10.1038/s41420-025-02338-7

**Published:** 2025-02-05

**Authors:** Jung Ha Kim, Semun Seong, Kabsun Kim, Inyoung Kim, Jeong Woo Park, Jeong-Tae Koh, Nacksung Kim

**Affiliations:** 1 Department of Pharmacology, Chonnam National University Medical School, Gwangju, 61469 Republic of Korea; 2 Hard-Tissue Biointerface Research Center, School of Dentistry, Chonnam National University, Gwangju, 61186 Republic of Korea; 3 Department of Biological Sciences, University of Ulsan, Ulsan, 44610 Republic of Korea; 4 Department of Pharmacology and Dental Therapeutics, School of Dentistry, Chonnam National University, Gwangju, 61186 Republic of Korea

**Keywords:** Cell signalling, Molecular biology

## Abstract

Multiple small GTPases play crucial roles in bone homeostasis by regulating the differentiation and function of bone cells, including osteoclasts and osteoblasts. Here, we investigated whether developmentally regulated GTP-binding protein 2 (Drg2), a subfamily of the GTPase superfamily, could affect bone mass by regulating osteoclast and osteoblast differentiation. Downregulation of Drg2 using siRNA in bone marrow-derived macrophages inhibited osteoclast differentiation and function and Rac1 activation in vitro. Comparatively, Drg2 downregulation in calvarial-derived osteoprogenitor cells enhanced osteoblast differentiation and function in vitro. Rac1 activation was also suppressed by Drg2 downregulation in osteoprogenitor cells. Both osteoclast and osteoblast differentiation regulated by Drg2 downregulation were restored by suppressing Rac1 activity. Drg2-deficient mice showed increased bone mass due to a dramatic reduction in osteoclast numbers without significantly affecting the number of osteoblasts. Furthermore, Drg2 downregulation strongly inhibited RANKL-induced bone loss in vivo. In summary, Drg2 contributes to bone homeostasis by regulating the differentiation and function of osteoclasts and osteoblasts through Rac1 activation. In particular, the effect of Drg2 on osteoclasts is strong enough to regulate bone mass in vivo; therefore, Drg2 has significant potential for use as a therapeutic target in bone loss-related diseases.

## Introduction

Small GTPases in the Ras superfamily, including the Ras, Rho, Arf, Ran, and Rab subfamilies, are key regulators of diverse cellular events, including cell division, vesicle transport, nuclear assembly, and cytoskeleton control [1]. Developmentally regulated GTP-binding proteins (Drgs) are novel GTPase members in the Ras superfamily and consist of two closely related proteins, Drg1 and Drg2 [2], which are broadly expressed in many human and mouse tissues [3]. Drg1 and Drg2 proteins are highly similar, sharing 55% amino acid sequence identity. Additionally, Drg1 and Drg2 have conserved binding partners: DRG family regulatory protein 1 and 2 (DFRP1 and DFRP2), respectively. Though Drg1 and Drg2 are highly similar, their respective binding partners share only limited similarity, suggesting that they have distinct functions [2].

Drg2 has been suggested to play a key role in cell cycle regulation during cell growth and differentiation, including mitochondrial function, and can affect cell migration by regulating microtubule dynamics and Golgi fragmentation [4, 5]. In particular, a previous study has shown that transgenic mice overexpressing Drg2 exhibited decreased bone mass and increased osteoclast numbers and activity in vivo [3]. Furthermore, the same study showed that the function of Drg2 in osteoclasts is associated with Rac1 activity [3]. Conversely, another previous study has demonstrated that 1-day-old Drg2-deficient mice exhibited significantly reduced mineralization of the skull and increased unossified areas in the anterior fontanel [4]. These results suggested that Drg2 may play a critical role in bone remodeling by regulating bone resorption and formation. However, previous studies on the bone phenotypes shown in transgenic mice overexpressing Drg2 and Drg2-deficient mice are controversial in explaining the function of Drg2 in bone remodeling. Therefore, although the physiological function of Drg2 in bone remodeling is very important, it remains limited and requires further investigation.

Bone remodeling is traditionally considered to consist of four sequential stages: the activation phase, where preosteoclast precursor cells are recruited to the damaged bone surface; the resorption phase, during which mature osteoclasts absorb the damaged bone; the reversal phase, where osteoclasts die, and preosteoblast precursor cells are mobilized; the formation phase, where mature osteoblasts generate new bone matrix that subsequently mineralizes. Nearly all new bone formation occurs within distinct anatomical structures known as basic multicellular units (BMUs), which correspond to previously resorbed sites. Maintaining bone mass and integrity requires a precise balance between bone resorption and formation [4, 6]. However, disruption in this balance due to impaired regulation between osteoclast-mediated bone resorption and osteoblast-mediated bone formation can lead to abnormal bone remodeling, resulting in secondary forms of osteoporosis such as postmenopausal- and glucocorticoid-induced osteoporosis [7].

Osteoclasts are derived from hematopoietic stem cells (HSCs) and break down bone through acid secretion and proteolytic enzymes, such as cathepsin K, which degrades the organic components of the bone matrix and dissolves the mineralized bone matrix [7, 8]. Osteoblasts arise from the commitment of mesenchymal precursors to osteoprogenitor lineages and secrete an unmineralized organic matrix called osteoid. This is subsequently mineralized through the accumulation of calcium phosphate in the form of hydroxyapatite [7, 9].

Rac1 represents the Rho family member of GTPase that participates in bone remodeling by regulating osteoclastic bone resorption and osteoblastic bone formation. In osteoclasts, Rac1 regulates survival, migration, and actin organization to increase bone resorption capacity and also plays a part in differentiation [10]. Rac1 activity inhibition promoted a disruption in the actin rings, reduced resorptive activity, and caused retraction of rat osteoclasts [1]. Research on osteoblast differentiation by Rac1 remains limited. However, conflicting reports exist. One study showed that Rac1 inhibition promotes osteoblast differentiation upon BMP-2 stimulation, whereas another showed that the absence of Rac1 in preosteoblasts diminishes osteoblast differentiation and function [11, 12].

Despite conflicting reported results, the findings that Drg2 activates Rac1 in osteoclasts and that both overexpression and deficiency of Drg2 affected bone mass in vivo suggest that Drg2 may play an important role during bone remodeling. Therefore, we investigated whether Drg2 regulates osteoclast and osteoblast differentiation dependently or independently of Rac1 activation to contribute to bone remodeling.

## Results

### Drg2 positively regulates osteoclast differentiation in a Rac1 activation-dependent manner

**Fig. 1:**
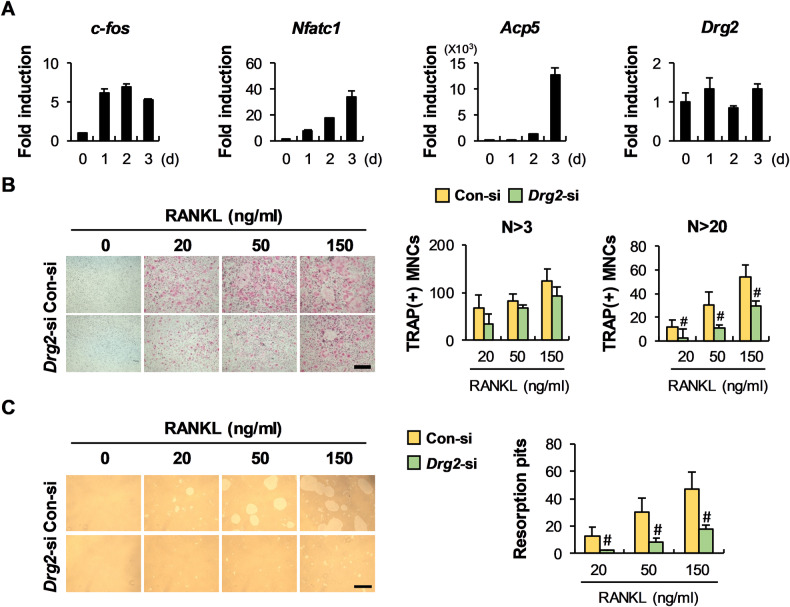
Downregulation of Drg2 in bone marrow-derived macrophages (BMMs) inhibits osteoclast differentiation. **A** BMMs were cultured in macrophage colony-stimulating factor (M-CSF) and receptor activator of nuclear factors κB ligand (RANKL) for the indicated times. The mRNA levels were analyzed using quantitative PCR. *N* = 3. **B** BMMs were cultured in M-CSF and RANKL for 3 days. The numbers of osteoclasts were determined after TRAP staining. *N* = 3. Scale bar, 200 µm. **C** BMMs were cultured in M-CSF and RANKL on the hydroxyapatite coating plate. The numbers of resorption pits were determined. *N* = 3. Scale bar, 200 µm. Data represent the mean ± SD of triplicate samples. #*P* < 0.05, *P < 0.01, **P < 0.001 vs. control.

**Fig. 2:**
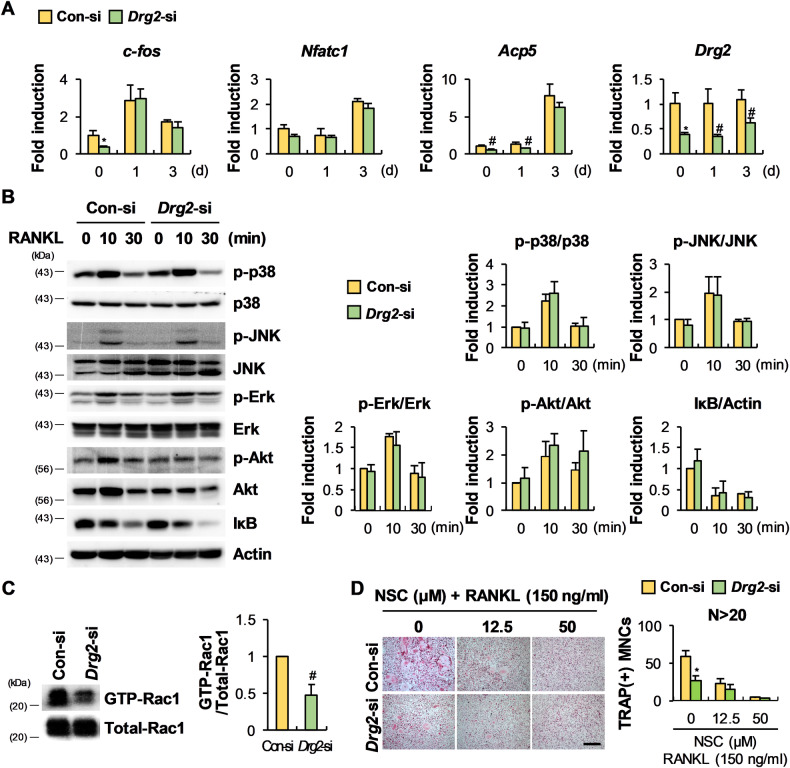
Inhibition of osteoclast differentiation by Drg2 downregulation in BMMs is associated with inhibition of Rac1 activation. **A** Control siRNA or Drg2 siRNA transfected-BMMs were cultured in M-CSF and RANKL for 3 days. The mRNA levels were analyzed using quantitative PCR. *N* = 3. **B** Control siRNA or Drg2 siRNA transfected-BMMs were serum-starved and stimulated using RANKL for the indicated times. Protein levels were assessed using Western immunoblotting. *N* = 3. **C** Control siRNA or Drg2 siRNA transfected-BMMs were stimulated with M-CSF and RANKL. Whole-cell lysates were incubated with GST fusion proteins containing the PAK1 RBD for the pulldown assay. Protein levels were assessed using Western immunoblotting. *N* = 3. **D** Control siRNA or Drg2 siRNA transfected-BMMs were cultured in M-CSF and RANKL in the absence or presence of NSC 23766. The numbers of osteoclasts were determined after TRAP staining. *N* = 3. Scale bar, 200 µm. Data represent the mean ± SD of triplicate samples. #*P* < 0.05, *P < 0.01 vs. control.

Drg2 is expressed in both osteoclast precursor cells and osteoclasts, and there was no noticeable change in expression during RANKL-induced osteoclast differentiation (Fig. 1A). To confirm the physiological role of Drg2 during osteoclast differentiation, Drg2 siRNA was transfected into osteoclast precursor cells. No significant difference was noted in the number of osteoclasts with more than three nuclei between the control- and Drg2-siRNA transfected cells; however, the number of osteoclasts with more than 20 nuclei was significantly inhibited by Drg2 downregulation (Fig. 1B). Moreover, inhibition of osteoclast formation with more than 20 nuclei following Drg2 downregulation resulted in decreased bone resorption (Fig. 1C). We next investigated the effects of Drg2 downregulation on osteoclast precursor cells relating to the expressions of osteoclast-related genes, the signaling pathways activated by RANKL, and Rac1 activation to elucidate the mechanism through which Drg2 regulates osteoclast differentiation. Drg2 downregulation had little effect on the expression of osteoclast-related genes such as *c-fos*, *Nfatc1*, and *Acp5* during RANKL-induced osteoclast differentiation (Fig. 2A). In osteoclast precursor cells, signaling pathways activated (p38, JNK, Erk, and Akt) and degraded (IκB) by RANKL stimulation remained unchanged following Drg2 downregulation (Fig. 2B). However, Rac1 activation was inhibited by Drg2 downregulation (Fig. 2C). Drg2 downregulation inhibited the formation of osteoclasts with more than 20 nuclei in the absence of NSC 23766, a potent Rac1 inhibitor; however, Drg2 downregulation did not significantly inhibit the formation of giant osteoclasts in the presence of NSC 23766, compared to the control (Fig. 2D). Collectively, these results indicate that Drg2 increases the formation of mature osteoclasts through Rac1 activation.

### Drg2 negatively regulates osteoblast differentiation in a Rac1 activation-dependent manner

**Fig. 3:**
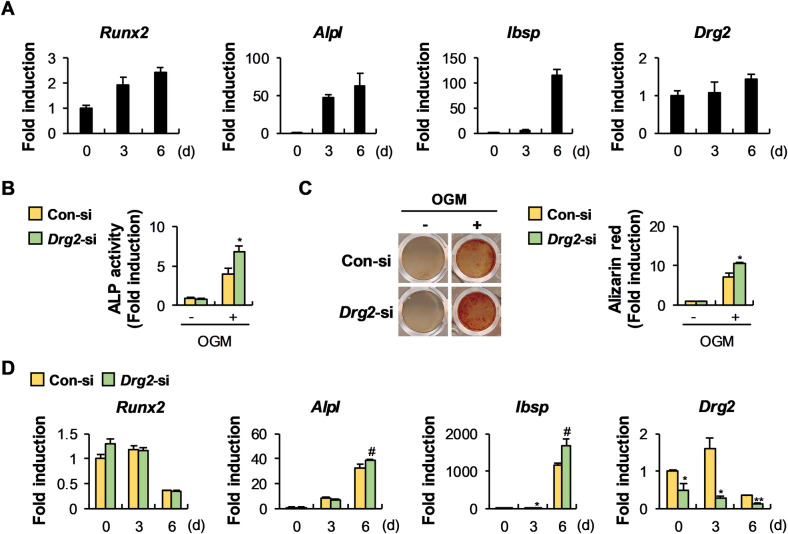
Downregulation of Drg2 in calvarial-derived osteoprogenitor cells promotes osteoblast differentiation and nodule formation. **A** Osteoprogenitor cells were cultured in an osteogenic medium (OGM) for the indicated times. The mRNA levels were analyzed using quantitative PCR. *N* = 3. **B** Control siRNA or Drg2 siRNA-transfected osteoprogenitor cells were cultured in OGM for 3 days. Cells were lysed, and alkaline phosphatase (ALP) activity was measured. *N* = 3. **C** Control siRNA or Drg2 siRNA-transfected osteoprogenitor cells were cultured in OGM for 6 days. Cells were stained with alizarin red and quantified via extraction. *N* = 3. **D** Control siRNA or Drg2 siRNA-transfected osteoprogenitor cells were cultured in OGM for the indicated times. The mRNA levels were analyzed using quantitative PCR. *N* = 3. Data represent the mean ± SD of triplicate samples. #*P* < 0.05, *P < 0.01 vs. control.

**Fig. 4:**
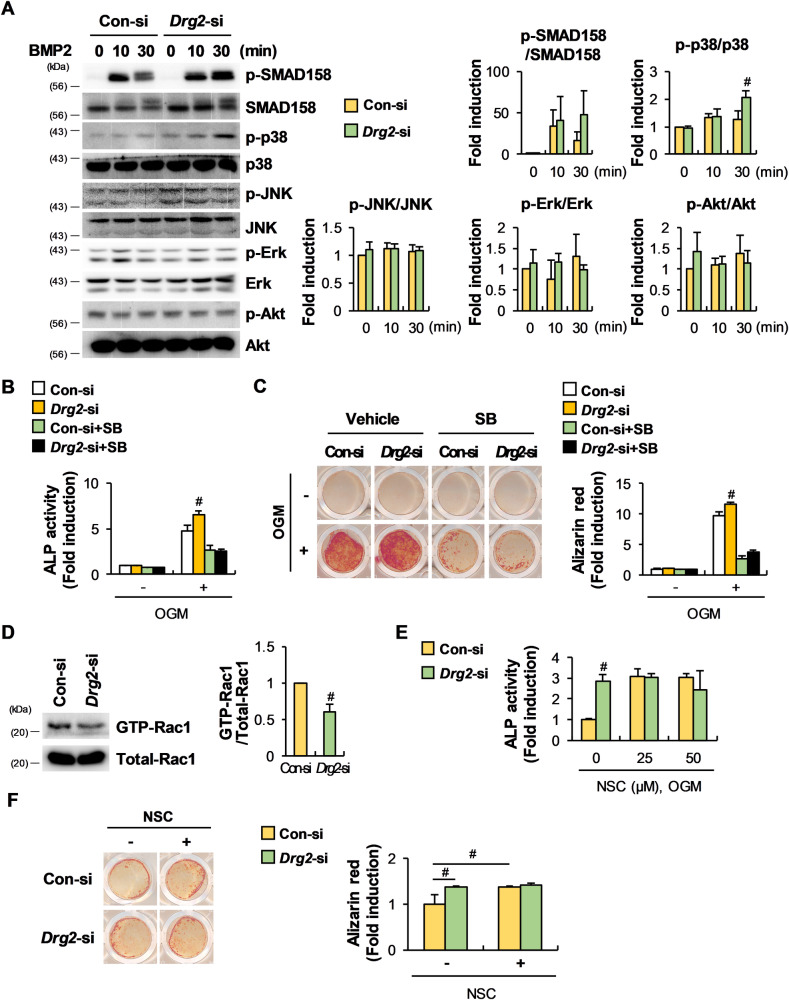
The stimulatory effect of Drg2 downregulation on osteoblast differentiation is associated with inhibition of Rac1 activation and increased p38 phosphorylation. **A** Control siRNA or Drg2 siRNA-transfected osteoprogenitor cells were serum-starved and stimulated using bone morphogenetic protein 2 (BMP2) for the indicated times. Protein levels were assessed using Western immunoblotting. *N* = 3. **B** Control siRNA or Drg2 siRNA-transfected osteoprogenitor cells were cultured in OGM in the absence or presence of SB253580 for 3 days. Cells were lysed, and ALP activity was measured. *N* = 3. **C** Control siRNA or Drg2 siRNA-transfected osteoprogenitor cells were cultured in OGM in the absence or presence of SB253580 for 6 days. Cells were stained with alizarin red and quantified via extraction. *N* = 3. **D** Control siRNA or Drg2 siRNA-transfected osteoprogenitor cells were stimulated with BMP2. Whole-cell lysates were incubated with GST fusion proteins containing the PAK1 RBD for the pulldown assay. Protein levels were assessed using Western immunoblotting. *N* = 3. **E** Control siRNA or Drg2 siRNA-transfected osteoprogenitor cells were cultured in OGM in the absence or presence of NSC 23766 for 3 days. Cells were lysed, and ALP activity was measured. *N* = 3. **F** Control siRNA or Drg2 siRNA-transfected osteoprogenitor cells were cultured in OGM in the absence or presence of NSC 23766 for 6 days. Cells were stained using alizarin red and quantified via extraction. *N* = 3. Data represent the mean ± SD of triplicate samples. #*P* < 0.05 vs. control.

Drg2 was consistently expressed during osteoblast differentiation (Fig. 3A). To determine the physiological role of Drg2 during osteoblast differentiation, Drg2 siRNA was transfected into osteoprogenitor cells. ALP activity and nodule formation were significantly increased in Drg2-siRNA transfected osteoprogenitor cells compared to controls (Fig. 3B, C). Consistent with these results, mRNA expression levels of osteoblast-related genes such as *Alpl* and *Ibsp* were significantly increased in osteoprogenitor cells transfected with Drg2 siRNA, compared to the controls (Fig. 3D). We next investigated the effects of Drg2 downregulation in osteoprogenitor cells on the activation of signaling pathways-related to osteoblast differentiation and Rac1 activation to elucidate the mechanism through which Drg2 regulates osteoblast differentiation. Among the examined signaling pathways, which included SMAD, p38, JNK, Erk, and Akt, p38 phosphorylation was significantly increased by Drg2 downregulation (Fig. 4A). Drg2 downregulation increased both ALP activity and nodule formation in the absence of SB203580, a potent p38 MAPK inhibitor. However, Drg2 downregulation failed to significantly increase either ALP activity or nodule formation in the presence of SB203580 (Fig. 4B, C). Interestingly, similar to osteoclasts, Rac1 activation was suppressed in osteoblasts by Drg2 downregulation (Fig. 4D). Both ALP activity and nodule formation were significantly increased by NSC 23766, and the increased effects of Drg2 downregulation on ALP activity and nodule formation were all eliminated by NSC 23766. These results suggest that Drg2 inhibits osteoblast differentiation by inhibiting p38 phosphorylation and promoting Rac1 activation.

### Osteoblast-dependent osteoclast formation is not affected by Drg2

**Fig. 5:**
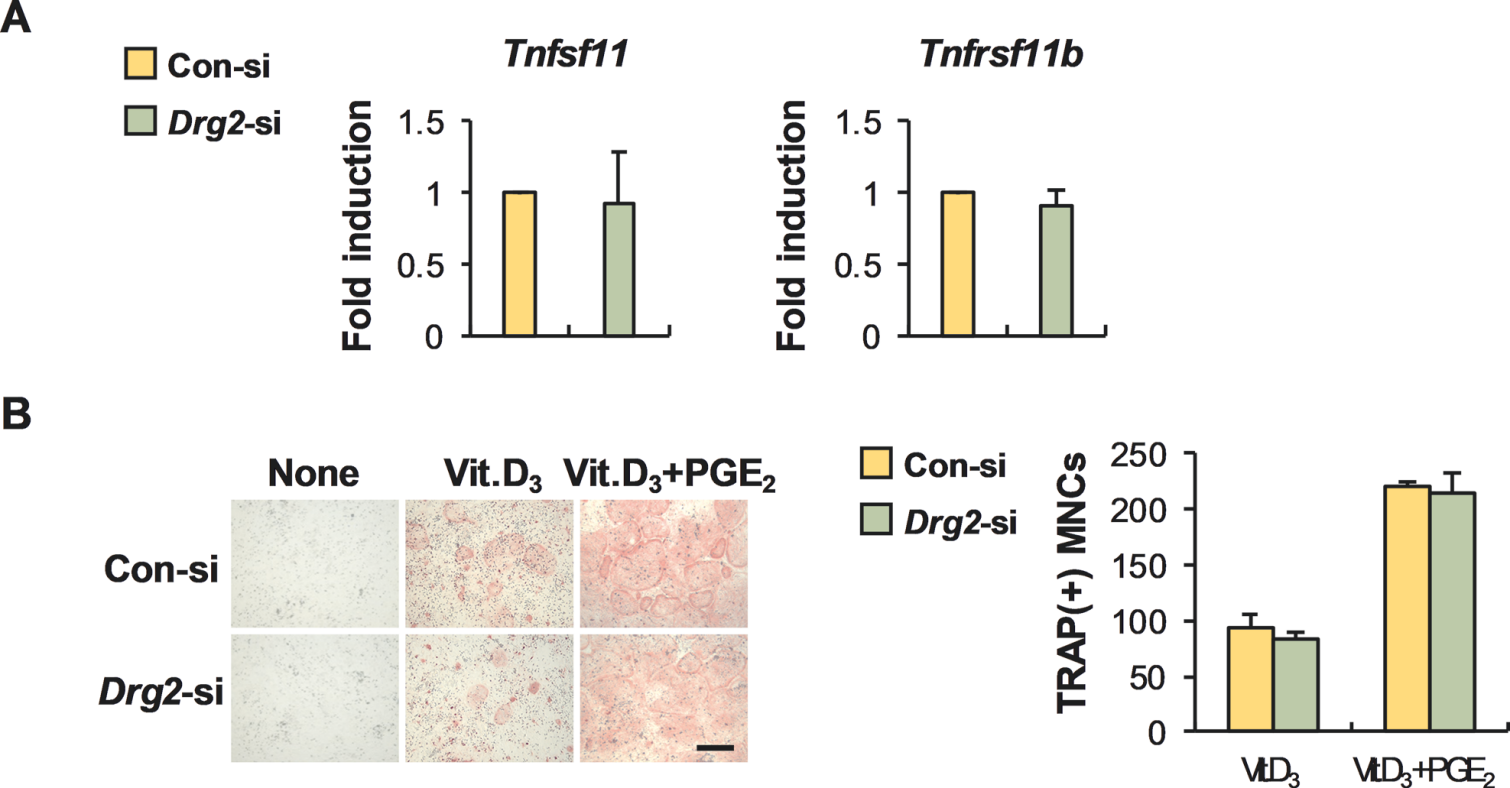
Drg2 does not affect the expression of RANKL (*Tnfsf11*) and osteoprotegerin (OPG, *Tnfrsf11b*). **A** Control siRNA or Drg2 siRNA-transfected osteoprogenitor cells were cultured in vitamin D3 (Vit.D_3_) for 2 days. The mRNA levels were analyzed using quantitative PCR. *N* = 3. **B** Control siRNA or Drg2 siRNA-transfected osteoprogenitor cells were co-cultured with BMMs in the presence or absence of vitamin D3 (Vit.D_3_) or prostaglandin E2 (PGE_2_) for 6 days. The numbers of osteoclasts were determined after TRAP staining. *N* = 3. Scale bar, 200 µm.

The cross-talk between osteoclasts and osteoblasts via cell–cell contact and secretory factors is essential for fine-tuning bone remodeling during bone homeostasis [7]. One important communication between osteoclasts and osteoblasts is that osteoblasts support osteoclast differentiation by regulating RANKL and osteoprotegerin (OPG) expressions. Therefore, we investigated the effect of Drg2 on RANKL and OPG expressions in osteoprogenitor cells to determine whether Drg2 is involved in supporting osteoclast differentiation by osteoblasts. As shown in Fig. 5A, Drg2 downregulation in osteoprogenitor cells did not regulate RANKL (*Tnfsf11*) or OPG (*Tnfrsf11b*) expression. As a result, when osteoclast precursor cells and osteoprogenitor cells were co-cultured, osteoclast formation was comparable between the control siRNA-transfected osteoprogenitor cells and Drg2 siRNA-transfected osteoprogenitor cells (Fig. 5B).

### Drg2 deficiency suppresses osteoclast differentiation and increases osteoblast differentiation in vitro

**Fig. 6:**
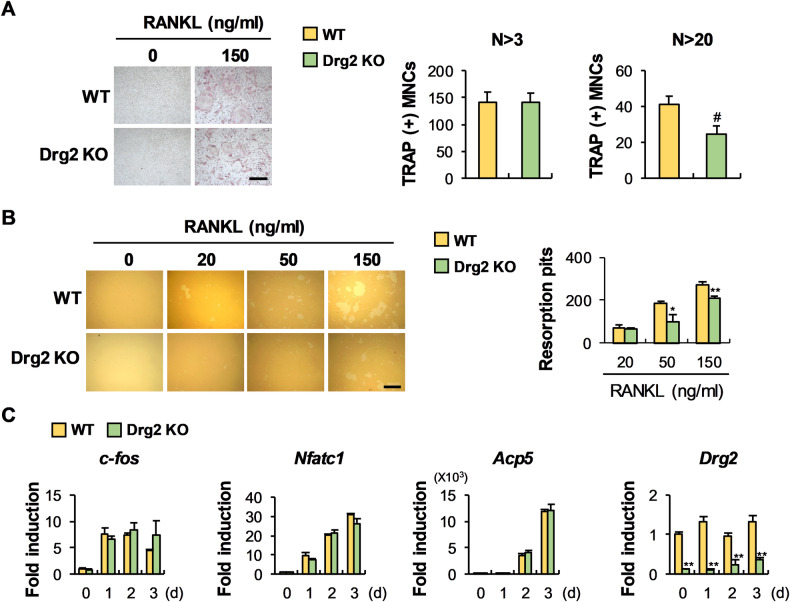
Drg2 deficiency inhibits osteoclast differentiation and function. **A** BMMs derived from wild-type or Drg2 knockout mice were cultured in M-CSF and RANKL. The osteoclast numbers were determined after TRAP staining. *N* = 3. Scale bar, 200 µm. **B** BMMs were cultured in M-CSF and RANKL on the hydroxyapatite coating plate. The numbers of resorption pits were determined. *N* = 3. Scale bar, 200 µm. **C** BMMs derived from wild-type or Drg2 knockout mice were cultured in M-CSF and RANKL for the indicated times. The mRNA levels were analyzed using quantitative PCR. Data represent the mean ± SD of triplicate samples. #*P* < 0.05, **P < 0.001 vs. control.

**Fig. 7:**
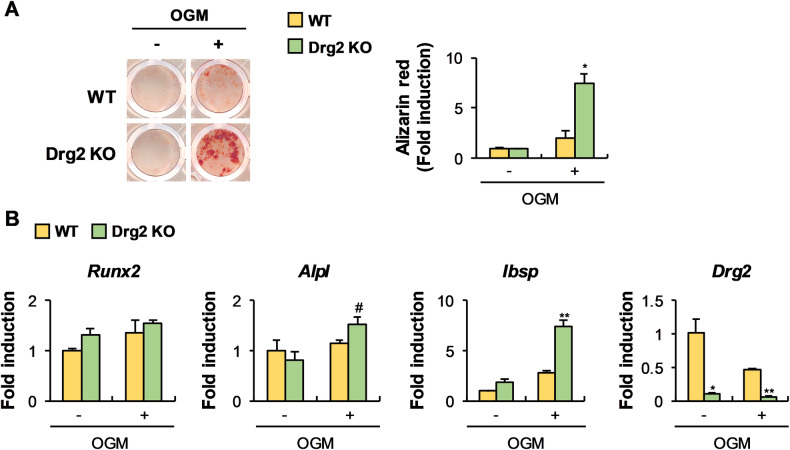
Drg2 deficiency promotes nodule formation. **A** Bone marrow stromal cells (BMSCs) from wild-type or Drg2 knockout mice were cultured in OGM. Cultured cells were stained using alizarin red and quantified via extraction. *N* = 3. **B** BMSCs from wild-type or Drg2 knockout mice were cultured in OGM. The mRNA levels were analyzed using quantitative PCR. Data represent the mean ± SD of triplicate samples. #*P* < 0.05, *P < 0.01, **P < 0.001 vs. control.

The physiological function of Drg2 on osteoclast and osteoblast differentiation was reconfirmed in Drg2-deficient cells derived from Drg2 knockout mice. The formation of osteoclasts with more than 20 nuclei was decreased in Drg2 knockout mice-derived osteoclast precursor cells compared to wild-type mice-derived osteoclast precursor cells (Fig. 6A), and the bone resorption capacity also followed a similar trend (Fig. 6B). Furthermore, no significant differences were observed in the expression of osteoclast-related genes such as *c-fos*, *Nfatc1*, and *Acp5* between osteoclast precursor cells derived from wild-type mice and Drg2 knockout mice (Fig. 6C), which is consistent with the Drg2 downregulation results. To determine the effect of Drg2 deficiency on osteoblast differentiation, bone marrow stromal cells (BMSCs) derived from wild-type mice and Drg2 knockout mice were differentiated into osteoblasts. As shown in Fig. 7A, nodule formation was increased in the BMSCs from Drg2 knockout mice compared to wild-type BMSCs. The expressions of *Alpl* and *Ibsp* were significantly increased in the BMSC derived from the Drg2 knockout mice compared to wild-type BMSCs (Fig. 7B). Collectively, these results show that Drg2 deficiency inhibits osteoclast differentiation and promotes osteoblast differentiation.

### Drg2 deficiency contributes to increased bone mass in vivo

**Fig. 8:**
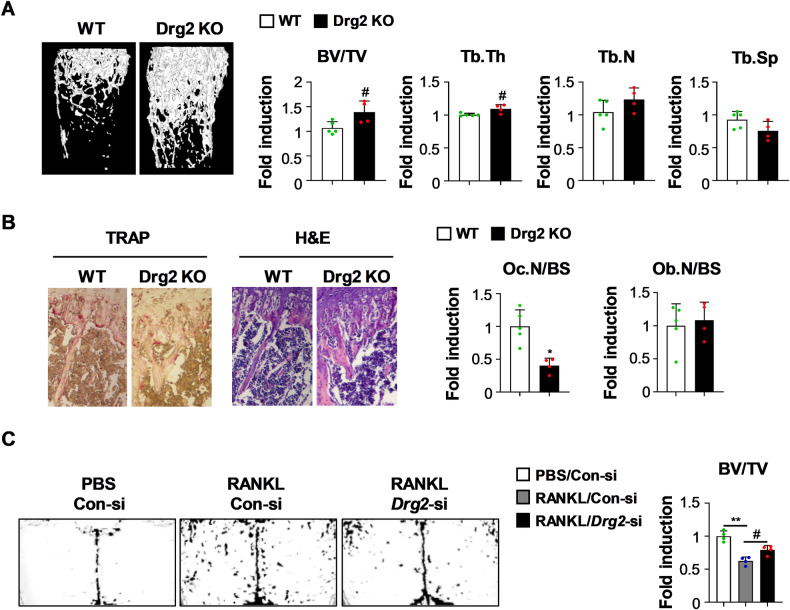
Drg2 knockout mice show slightly increased bone mass. **A** Femurs from wild-type or Drg2 knockout mice were subjected to micro-computed tomography. Representative 3D images of femurs were analyzed. Bone volume/tissue volume (BV/TV), trabecular thickness (Tb.Th), trabecular number (Tb.N), and trabecular separation (Tb.Sp) were determined. (*N* = 5 or 4). **B** Tibias from wild-type or Drg2 knockout mice were subjected to histological analyses. Images of tibiae stained using TRAP and H&E were analyzed, and the number of osteoclasts and osteoblasts was quantified. (*N* = 5 or 4). **C** Control siRNA or Drg2 siRNA was injected into mouse calvaria one day before collagen sponge implantation. Collagen sponges soaked with PBS or RANKL were implanted into mouse calvaria. Control siRNA or Drg2 siRNA was injected into mouse calvaria every 2 days for 5 days. Bone volume/tissue volume (BV/TV) was analyzed using micro-computed tomography. (*N* = 4). Data represent the mean ± SD of triplicate samples. #*P* < 0.05, *P < 0.01, **P < 0.001 vs. control.

Next, we analyzed the bone phenotypes of Drg2 knockout mice and whether Drg2 deficiency affects bone mass in vivo by inhibiting osteoclast differentiation and promoting osteoblast differentiation. Drg2 knockout mice showed significantly increased trabecular bone volume and trabecular thickness, slightly increased trabecular numbers, and slightly decreased trabecular separation, resulting in increased bone mass compared to wild-type mice (Fig. 8A). Moreover, the number of osteoclasts was significantly reduced in the Drg2 knockout mice compared to the wild-type mice, whereas, unexpectedly, the number of osteoblasts was only slightly increased compared to wild-type mice (Fig. 8B). Moreover, Drg2 downregulation alleviated bone loss induced following RANKL administration into the calvaria of mice (Fig. 8C). Overall, these results suggest that Drg2 can regulate bone mass by strongly influencing osteoclasts under physiological conditions; further, that Drg2 can be used as a therapeutic target when bone mass is pathologically reduced.

## Discussion

Small GTPases are one of the most important regulators of signal transduction in various cell types. Substantial evidence has been reported that multiple small GTPases are also crucial for osteoclast function. Rho was initially studied in osteoclasts; subsequently, small GTPases such as Rac, RhoU, Cdc42, ARF6, and Rab7 have been reported to play an important role in osteoclast differentiation, function, and survival [13]. Since small GTPases all require prenylation post-translation modifications for their correct localization and function, drugs that can inhibit prenylation of small GTPases are most widely used to inhibit bone loss by osteoclasts in common metabolic diseases [13]. In particular, Rac1 was reported to be crucial for osteoclast differentiation and function without affecting the transcription of genes essential for osteoclast differentiation, such as *c-fos*, *c-Src*, and *Nfatc1* [14, 15]. In this study, we further clarified Drg2 as a small GTPase important for osteoclast differentiation and function. The role of Drg2 in osteoclasts was similar to Rac1. Drg2 was involved in forming mature osteoclasts without affecting either RANKL-activated early signaling pathways or RANKL-induced expression of osteoclast-related genes, which resulted in bone resorption. Furthermore, the role of Drg2 in osteoclasts was associated with Rac1 activation. Therefore, Drg2 is one of the small GTPases contributing to Rac1 activation to form mature osteoclasts.

The function of small GTPases in osteoblasts remained largely unexplored compared to osteoclasts. Previous studies have reported the important roles of a few small GTPases in the differentiation and function of osteoblasts. For example, it was reported that CDC42 enhances the differentiation of adult MSCs into osteoblasts, genetic depletion of intraflagellar transport protein 80 (IFT80) reduces the expression of Runx2 during differentiation of murine MSCs into osteoblasts, and direct inhibition of RhoA upregulates zolendronic acid-induced BSP expression [16–18]. Conflicting results have been reported regarding the function of Rac1 in osteoblasts. While it was found that Rac1 deletion suppresses differentiation in preosteoblasts but not in differentiating osteoblasts, it was also reported that Rac1 inhibition promotes osteoblast differentiation upon BMP2 stimulation [11, 12, 14]. This study showed that Drg2 is involved in Rac1 activation in osteoblasts and osteoclasts. Moreover, Drg2 downregulation in osteoprogenitor cells inhibited Rac1 activation and increased osteoblast differentiation. Meanwhile, the effect of promoting osteoblast differentiation caused by Drg2 downregulation was abolished by inhibiting Rac1 activity. Therefore, Drg2 and Rac1 act repressively on osteoblast differentiation, and the impact of Drg2 on osteoblast differentiation depends on Rac1 activation. However, since it has been shown that Drg2 also regulates osteoblast differentiation through p38 phosphorylation, it seems that the action of Drg2 in osteoblasts is not entirely through Rac1 activation.

To examine the physiological function of Drg2 in bone metabolism in vivo, we analyzed bone mass, osteoclasts, and osteoblasts in the femurs of Drg2 knockout mice. Inhibition of osteoclast differentiation and promoting osteoblast differentiation by Drg2 deficiency in vitro led to the prediction that bone mass would dramatically increase in vivo. However, bone mass increased slightly in the Drg2 knockout mice compared with the wild-type mice. Furthermore, the increase in bone mass was associated with significant increases in trabecular bone volume and trabecular thickness and a non-significant mild increase and decrease in trabecular number and separation. The mild changes in the bone phenotype of Drg2 knockout mice were unexpectedly found to be due to osteoblast numbers being similar to wild-type, despite a strong reduction in osteoclast numbers in Drg2 knockout mice. Two previous studies suggested an effect of Drg2 on bone mass in vivo [3, 4]. Ke et al. showed that transgenic mice overexpressing Drg2 had decreased bone density due to increased osteoclast-mediated bone resorption [3]. Conversely, Lim et al. showed that micro-CT analyses of Drg2-deficient mice revealed a significant increase in the unossified area in the skull and a dramatic reduction in bone formation in the appendicular skeleton [4]. Analysis of femurs from 10-week-old mice by colleagues of ours and Ke identified the effect of Drg2 on bone-resorbing osteoclasts but not on bone-forming osteoblasts. In contrast, Lim and colleagues confirmed the skeletons of 1-day-old mice using only micro-CT without analyzing osteoclasts and osteoblasts in vivo. Therefore, the discrepancy between studies assessing the effect of Drg2 on bone mass, including our current results, may result from the complexity of the impact of Drg2 on bone formation during different developmental stages. Subsequently, it is necessary to consider the roles of Rac1 in bone formation by osteoblasts. When we confirmed the inhibitory effect of Rac1 activation on osteoblast differentiation in vitro, we did not examine it in osteoblastic cells at various stages of differentiation but only in osteoprogenitor cells. Therefore, we cannot rule out the possibility that the effects of Rac1 may differ depending on the stage of osteoblast differentiation. Moreover, existence of conflicting reports regarding the roles of Rac1 in osteogenesis and the findings that there is no significant change in osteogenesis in 10-week-old mice overexpressing Drg2 or those with a Drg2 deficiency suggest the possibility that Rac1 may have different functions in osteogenesis depending on the various developmental stages or target cells (mesenchymal stem cells, osteoprogenitors, preosteoblasts, or osteoblasts) in vivo. One more important point to consider as a possible cause of the weak effect of Drg2 deficiency on osteoblast numbers in vivo is that maintaining bone mass and integrity requires a precise balance between bone resorption and formation. The strong reduction in the number of osteoclasts in Drg2 knockout mice may cause a decrease in the differentiation and function of osteoblasts to maintain bone homeostasis.

In this study, we clarified that Drg2 deficiency can inhibit bone resorption in vivo, although the effect of Drg2 deficiency on osteogenesis in vivo remains to be explained. These results suggest modulating Drg2 expression may be a pharmacological approach to alleviate excessive osteoclast activity and bone loss under certain pathological conditions. This is supported by the current results showing that Drg2 downregulation alleviated bone loss in a mouse model where RANKL promoted bone loss.

In summary, Drg2 increases osteoclast differentiation through Rac1 activation, increasing bone resorption in vitro. Drg2 reduces osteoblast differentiation through Rac1 activation and inhibition of p38 phosphorylation in vitro. Drg2 deficiency increases bone mass by strongly inhibiting osteoclast-mediated bone resorption without affecting osteoblast-mediated bone formation in vivo. Further research on the role of Drg2 in bone formation in vivo could establish it as a therapeutic target for bone diseases.

## Material and methods

### Mice

Drg2 knockout mice were generated by Macrogen (Seoul, Korea), as previously described [19]. Briefly, a targeting vector was designed to replace Drg2 exons 1 and 2 with a PGK-neo cassette using 5’ (2.6 kb) and 3’ (7.3 kb) arm fragments of Drg2 ligated into the pPNT vector. The mice were genotyped via PCR analysis of tail snip genomic DNA with the following primers: GAG-CGG-AGG-TGA-TGA-GAG-TCA-AGA-G, AGC-ACG-TAC-TCG-GAT-GGA-AGC-CGG-TC, and ACT-CTG-GCA-ACT-CTC-TGC-AAC-AAC-A.

### Osteoclast differentiation and resorption analysis

Bone marrow cells (BMCs) were isolated from the femurs and tibias of ICR mice and cultured in α-MEM containing 10% FBS and M-CSF (30 ng/mL) for 3 days to generate bone marrow-derived macrophages (BMMs), which serve as osteoclast precursors. These BMMs were then further cultured in α-MEM supplemented with 10% FBS, M-CSF (30 ng/mL), and RANKL (20–150 ng/mL). The cultured cells were fixed using 3.7% formalin and treated with tartrate-resistant acid phosphatase (TRAP) staining solution (Sigma-Aldrich, St Louis, MO) for 10 minutes. Images of the stained cells were captured using a ProRes CFscan camera (Jenoptik, Jena, Germany) and analyzed using ProgRes Capture Pro software. For the resorption analysis, BMMs were cultured in Osteo assay plates (Corning, Corning, NY) in the presence of M-CSF (30 ng/mL) and RANKL (20–100 ng/mL) for 3 days. Resorption pits were visualized under bright-field microscopy, and the number of resorption pits per well was counted. For co-culture experiments, BMMs were co-cultured with osteoblasts in the presence of vitamin D3 (10^−8^ M) or vitamin D3 (10^−8^ M) and prostaglandin E2 (10^−7^ M) for 6 days. The cultured cells were then fixed and stained using TRAP solution.

### Osteoblast differentiation and nodule formation

Neonatal mouse calvariae was enzymatically digested to obtain osteoprogenitor cells. The calvariae were treated five times with 0.1% collagenase (Life Technologies, Carlsbad, CA) and 0.2% dispase II (Roche Diagnostics Gmbh, Mannheim, Germany) at 37 °C for 10 min. The first cell set was discarded, and the second to fifth sets of cells were collected. Osteoblast differentiation was induced by culture medium supplemented with 100 ng/mL BMP2 (Cowellmedi, Busan, Korea), 50 μg/mL ascorbic acid (Sigma-Aldrich), and 10 mM β-glycerophosphate (Sigma-Aldrich). After culturing for 3 days, the cultured cells were lysed. Then, cell lysates were incubated with p-nitrophenyl phosphate substrate (Sigma-Aldrich, St Louis, MO) to measure ALP activity by measuring the absorbance at 405 nm. The cells cultured for 6 days were fixed using 3.7% formalin and stained using 40 mM alizarin red (pH 4.2) for 10 min. The stained culture plates were scanned using a CanoScan 9900F (Canon Inc., Japan). The stained cells were treated using 10% acetic acid to extract alizarin red, and the absorbance at 405 nm was measured to quantify the degree of matrix mineralization.

### Western blotting

Cultured cells were lysed to extract proteins, and equal amounts of proteins were separated using sodium dodecyl sulfate-polyacrylamide gel electrophoresis and subsequently transferred to a polyvinylidene fluoride membrane (Millipore, Billerica, MA). The antibodies used are listed as follows: actin (Sigma-Aldrich), phosphor-p38, p38, phosphor-JNK, JNK, phosphor-AKT, AKT, phosphor-SMAD158, SMAD158, and IκB (Cell Signaling Technology, Beverly, MA). Activity assays for Rac1 were measured using small GTPase activation kits (Pierce/Thermo Scientific, Rockford, IL) according to the manufacturer’s protocol. Briefly, GTP-bound Rac1 was pulled-down from whole-cell extracts using GST-fused PAK1 CRIB domain. SDS-PAGE analyzed affinity precipitates and whole-cell lysates, and Rac1 was detected.

### Real-time PCR

Total RNA was isolated from the cultured cells using Qiazol (Qiagen GmbH, Hilden, Germany) according to the manufacturer’s protocol. Reverse transcription was performed using a QuantiNova Reverse Transcription kit (Qiagen). Quantitative real-time PCR analyses were conducted on the Rotor-Gene Q (Qiagen). The comparative computed tomography method was used to evaluate the relative messenger RNA levels, and relative gene expression was normalized against *Gapdh*. The primers used are as follows (gene name, forward primer, reverse primer): *c-fos*, ATG-GGC-TCT-CCT-GTC-AAC-ACA-CAG, TGG-CAA-TCT-CAG-TCT-GCA-ACG-CAG; *Nfatc1*, CTC-GAA-AGA-CAG-CAC-TGG-AGC-AT, CGG-CTG-CCT-TCC-GTC-TCA-TAG; *Acp5*, TCC-GTG-CTC-GGC-GAT-GGA-CCA-GA, CTG-GAG-TGC-ACG-ATG-CCA-GCG-ACA; *Drg2*, CGC-ACA-GCT-GAT-GTC-GTC-GTC-ATG, GTC-TAC-TTC-TTC-CAT-GGA-GAT-CTG; *Runx2*, CCC-AGC-CAC-CTT-TAC-CTA-CA, CAG-CGT-CAA-CAC-CAT-CAT-TC; *Alpl*, CAA-GGA-TAT-CGA-CGT-GAT-CAT-G, GTC-AGT-CAG-GTT-GTT-CCG-ATT-C; *Ibsp*, GGA-AGA-GGA-GAC-TTC-AAA-CGA-AG, CAT-CCA-CTT-CTG-CTT-CTT-CGT-TC; *Tnfsf11*, CCT-GAG-ACT-CCA-TGA-AAA-CGC, TCG-CTG-GGC-CAC-ATC-CAA-CCA-TGA; *Tnfrsf11b*, CAG-TGA-TGA-GTG-TGT-GTA-TTG-CAG, TTA-TAC-AGG-GTG-CTT-TCG-ATG-AAG; *Gapdh*, TGA-CCA-CAG-TCC-ATG-CCA-TCA-CTG, CAG-GAG-ACA-ACC-TGG-TCC-TCA-GTG.

### RANKL-induced bone loss

Control siRNA or Drg2 siRNA (30 µL of 20 µM) was mixed with Lipofectamine RNAiMAX (10 µL; Invitrogen) and injected into the calvariae of mice. The following day, collagen sponges soaked in PBS or RANKL (2 mg/kg) were implanted into the calvariae of the mice. Control siRNA or Drg2 siRAN mixed with Lipofectamine RNAiMAX was injected into the calvariae of the mice every 2 days for a total of 5 days.

### Micro-CT analysis

Isolated distal femurs were scanned and analyzed using the SkyScan 1172 system (SkyScan, Kotich, Belgium). The scanning parameters included 50 kV and 201 μA with a 0.5 mm aluminum filter, resulting in a resolution of 11 μm per pixel. Images were captured at intervals of 0.7° over an angular range of 180°. Raw images were reconstructed into serial cross-sections and femoral morphometric parameters were analyzed using image reconstruction software (NRecon 1.4; SkyScan), data analysis software (CTAn; SkyScan), and a three-dimensional model visualization software (Ant 2.4; SkyScan).

### Histology and TRAP staining

Tibias were fixed using 4% paraformaldehyde, decalcified, dehydrated, and embedded in paraffin blocks. The tibias embedded in paraffin blocks were sectioned longitudinally at a thickness of 4 μm, deparaffinized using xylene, and then stained using TRAP and H&E.

### Statistical analyses

All values are expressed as the mean ± SD. Statistically significant differences were determined using a two-tailed Student’s *t* test for two independent samples. For statistical analyses involving multiple group comparisons, analysis of variance (ANOVA) with post-hoc Tukey HSD test was performed to calculate *P* values. Differences with a *P* value less than 0.05 were considered statistically significant.

## Supplementary information

Orginal WB

## Data Availability

All data and materials are included in this manuscript.
