# Supplementary material for: Rac1-dependent regulation of osteoclast and osteoblast differentiation by developmentally regulated GTP-binding 2
Source: Cell Death Discov. 2025 Feb 5;11:48. doi: 10.1038/s41420-025-02338-7 (PMC11799230; doi:10.1038/s41420-025-02338-7)
Supplement: Supplementary file 1 — Orginal WB [file 41420_2025_2338_MOESM1_ESM.pdf]

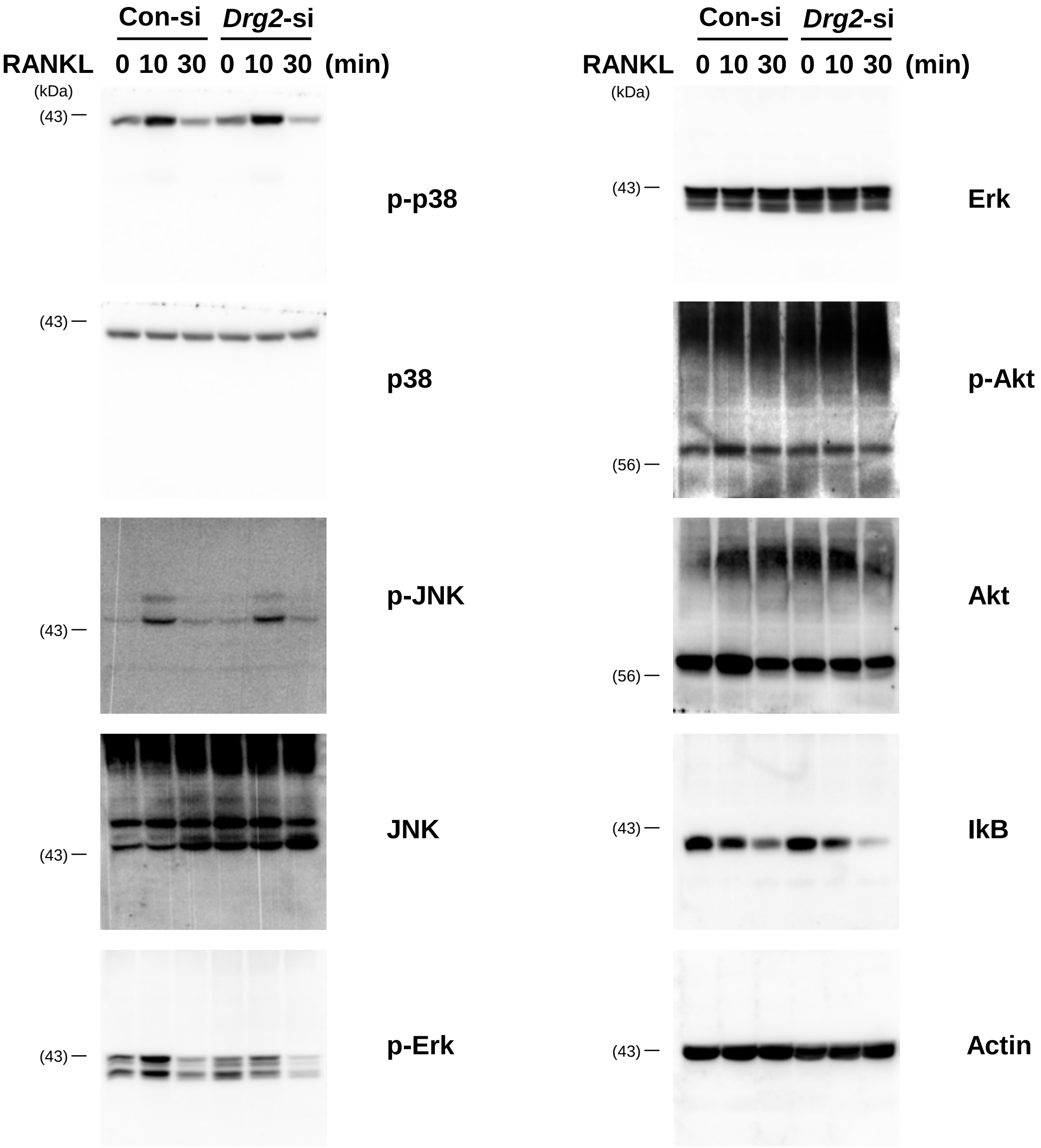

Fig. 2B original western blots

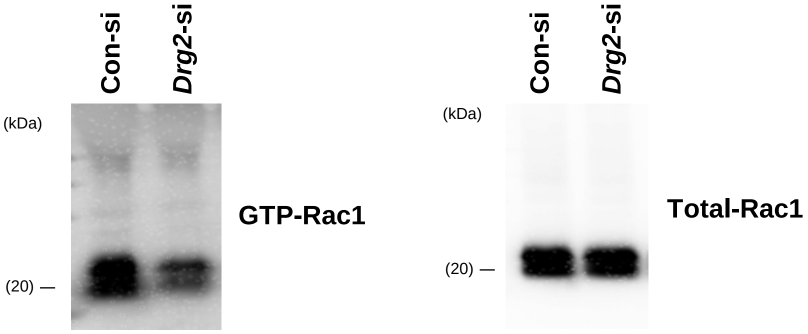

Fig. 2C original western blots

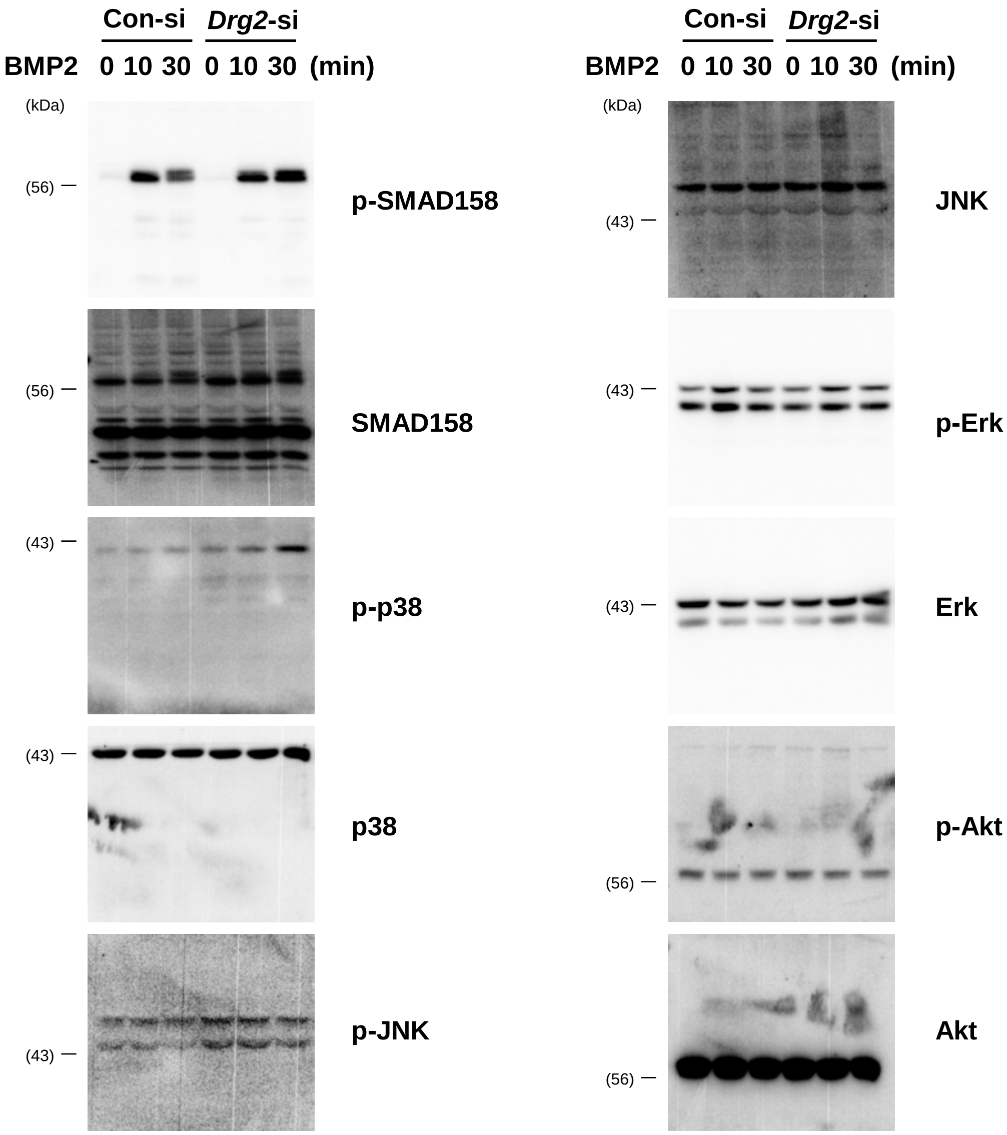

Fig. 4A original western blots

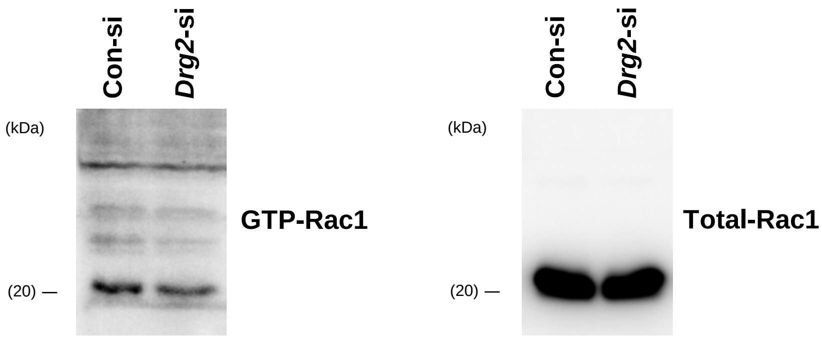

Fig. 4D original western blots
